# Supplementary material for: Evaluation of soil heavy metals pollution and the phytoremediation potential of copper-nickel mine tailings ponds
Source: PLoS One. 2023 Mar 3;18(3):e0277159. doi: 10.1371/journal.pone.0277159 (PMC9983881; doi:10.1371/journal.pone.0277159)
Supplement: Attachment — Submitted filename: PONE-D-22-29071.pdf [file pone.0277159.s002.pdf]

PLOS ONE

Heavy metal pollution characteristics of soil around a copper-nickel mine tailings pond in the northwest arid area of China and evaluation of desert phytoremediation potential

--Manuscript Draft--

Manuscript Number:	PONE-D-22-29071
Article Type:	Research Article
Full Title:	Heavy metal pollution characteristics of soil around a copper-nickel mine tailings pond in the northwest arid area of China and evaluation of desert phytoremediation potential
Short Title:	Evaluation of phytoremediation potential of heavy metal pollution in soil by desert plants
Corresponding Author:	Jianfei Shi<br>Xinjiang Institute of Ecology and Geography<br>Urumqi, Xinjiang province CHINA
Keywords:	Arid region; Tailings pond; Heavy metal pollution; Fuzzy evaluation; Desert plants; Phytoremediation
Abstract:	Phytoextraction and phytostabilization are two mechanisms used by plants to remove and stabilize heavy metals in soil. However, there has been little research on the extraction/stabilization of heavy metals by desert plants in arid areas. This study analyzed eight heavy metals (Cr, Ni, Cu, Zn, Cd, Pb, Mn, and As) in 10 desert plants and their growth matrices. In addition, the single factor pollution index and Nemerow comprehensive pollution index were used to evaluate soil pollution. Based on the biological accumulation factor and the biological concentration factor, the fuzzy evaluation method was used to evaluate plant extraction/stability potentials for heavy metals. The results showed that Cd, Cu, Ni, and Cr in the soil around the tailings pond were at the heavy pollution level, Mn and Pb pollution was moderate, and Zn and As pollution was light. The matrix factorization model showed that Cu and Ni came from industrial pollution; Cd and Cr came from atmospheric deposition and agricultural pollution; Pb came from traffic pollution; and Mn, Zn, and As came from natural sources. The metal contents of the desert plants exceeded the standard for normal plants, of which Cr contents in the Atriplex patens and Ammophila breviligulata Fernald aboveground parts were 35.63 and 53.77 mg/kg respectively, and the Ni contents in the Klasea centauroides subsp. polycephala (Iljin) L. Martins underground parts and the A. breviligulata Fernald aboveground parts were 102.67 and 101.67 mg/kg, respectively, exceeding the maximum toxicity threshold for Cr and Ni. Ammophila breviligulata Fernald had the highest plant comprehensive extraction coefficient (CEI) and plant comprehensive stability coefficient (CSI) at 0.81 and 0.83 respectively, indicating that it has strong heavy metal extraction and stabilization abilities. Therefore, A. breviligulata Fernald can be selected as a remediation species for heavy metal pollution in the soil around tailings reservoir areas.
Order of Authors:	Jianfei Shi
	Zhengzhong Jin
	Zhibin Zhou
	Xin Wang
	Xiaoliang Yang
	Wenting Qian
Additional Information:	
Question	Response
**Financial Disclosure**<br><br>Enter a financial disclosure statement that describes the sources of funding for the	This work was funded by the National Key Research and Development Program of China (2018YFC1802903).

Powered by Editorial Manager® and ProduXion Manager® from Aries Systems Corporation

work included in this submission. Review the submission guidelines for detailed requirements. View published research articles from *PLOS ONE* for specific examples.<br><br>This statement is required for submission and **will appear in the published article** if the submission is accepted. Please make sure it is accurate.<br><br>**Unfunded studies**<br>Enter: *The author(s) received no specific funding for this work.*<br><br>**Funded studies**<br>Enter a statement with the following details:<br>• Initials of the authors who received each award<br>• Grant numbers awarded to each author<br>• The full name of each funder<br>• URL of each funder website<br>• Did the sponsors or funders play any role in the study design, data collection and analysis, decision to publish, or preparation of the manuscript?<br>• **NO** - Include this sentence at the end of your statement: *The funders had no role in study design, data collection and analysis, decision to publish, or preparation of the manuscript.*<br>• **YES** - Specify the role(s) played.<br><br>* typeset	
**Competing Interests**<br><br>Use the instructions below to enter a competing interest statement for this submission. On behalf of all authors, disclose any competing interests that could be perceived to bias this work—acknowledging all financial support and any other relevant financial or non-financial competing interests.<br><br>This statement is required for submission and **will appear in the published article** if the submission is accepted. Please make sure it is accurate and that any funding sources listed in your Funding Information	The authors have declared that no competing interests exist.

later in the submission form are also declared in your Financial Disclosure statement.<br><br>View published research articles from *PLOS ONE* for specific examples.<br><br>**NO authors have competing interests**<br><br>Enter: *The authors have declared that no competing interests exist.*<br><br>**Authors with competing interests**<br><br>Enter competing interest details beginning with this statement:<br><br>*I have read the journal's policy and the authors of this manuscript have the following competing interests: [insert competing interests here]*<br><br>* typeset	
**Ethics Statement**<br><br>Enter an ethics statement for this submission. This statement is required if the study involved:<br><br>• Human participants<br>• Human specimens or tissue<br>• Vertebrate animals or cephalopods<br>• Vertebrate embryos or tissues<br>• Field research<br><br>Write "N/A" if the submission does not require an ethics statement.<br><br>General guidance is provided below. Consult the submission guidelines for detailed instructions. **Make sure that all information entered here is included in the Methods section of the manuscript.**	N/A

**Format for specific study types**<br><br>**Human Subject Research (involving human participants and/or tissue)**<br>• Give the name of the institutional review board or ethics committee that approved the study<br>• Include the approval number and/or a statement indicating approval of this research<br>• Indicate the form of consent obtained (written/oral) or the reason that consent was not obtained (e.g. the data were analyzed anonymously)<br><br>**Animal Research (involving vertebrate animals, embryos or tissues)**<br>• Provide the name of the Institutional Animal Care and Use Committee (IACUC) or other relevant ethics board that reviewed the study protocol, and indicate whether they approved this research or granted a formal waiver of ethical approval<br>• Include an approval number if one was obtained<br>• If the study involved *non-human primates*, add *additional details* about animal welfare and steps taken to ameliorate suffering<br>• If anesthesia, euthanasia, or any kind of animal sacrifice is part of the study, include briefly which substances and/or methods were applied<br><br>**Field Research**<br><br>Include the following details if this study involves the collection of plant, animal, or other materials from a natural setting:<br>• Field permit number<br>• Name of the institution or relevant body that granted permission	
**Data Availability**<br><br>Authors are required to make all data underlying the findings described fully available, without restriction, and from the time of publication. PLOS allows rare exceptions to address legal and ethical concerns. See the PLOS Data Policy and FAQ for detailed information.	Yes - all data are fully available without restriction

A Data Availability Statement describing where the data can be found is required at submission. Your answers to this question constitute the Data Availability Statement and **will be published in the article**, if accepted.<br><br>**Important:** Stating 'data available on request from the author' is not sufficient. If your data are only available upon request, select 'No' for the first question and explain your exceptional situation in the text box.<br><br>Do the authors confirm that all data underlying the findings described in their manuscript are fully available without restriction?	
**Describe where the data may be found in full sentences. If you are copying our sample text, replace any instances of XXX with the appropriate details.**<br><br>• If the data are **held or will be held in a public repository**, include URLs, accession numbers or DOIs. If this information will only be available after acceptance, indicate this by ticking the box below. For example: *All XXX files are available from the XXX database (accession number(s) XXX, XXX.).*<br>• If the data are all contained **within the manuscript and/or Supporting Information files**, enter the following: *All relevant data are within the manuscript and its Supporting Information files.*<br>• If neither of these applies but you are able to provide **details of access elsewhere**, with or without limitations, please do so. For example:<br><br>*Data cannot be shared publicly because of [XXX]. Data are available from the XXX Institutional Data Access / Ethics Committee (contact via XXX) for researchers who meet the criteria for access to confidential data.*<br><br>*The data underlying the results presented in the study are available from (include the name of the third party*	All relevant data are within the manuscript and its Supporting Information files

and contact information or URL).<br>• This text is appropriate if the data are owned by a third party and authors do not have permission to share the data.<br><br>* typeset	
Additional data availability information:	

Manuscript

Click here to access/download;Manuscript;manuscript_shiPO.docx

Heavy metal pollution characteristics of soil around a copper-nickel mine tailings pond in the northwest arid area of China and evaluation of desert phytoremediation potential

Jianfei Shi[a, b], Zhengzhong Jin[b], Zhibin Zhou[b], Xin Wang[a, b], Xiaoliang Yang[a, b], Wenting Qian[b, *]

aUniversity of Chinese Academy of Science, Beijing 100049, China

bXinjiang Institute of Ecology and Geography, Chinese Academy of Science/National Desert-Oasis Ecology Construction Engineering Technology Research Center, Urumqi 830054, China

*Corresponding author: Wenting Qian (qwt@ms.xjb.ac.cn)

ABSTRACT

Phytoextraction and phytostabilization are two mechanisms used by plants to remove and stabilize heavy metals in soil. However, there has been little research on the extraction/stabilization of heavy metals by desert plants in arid areas. This study analyzed eight heavy metals (Cr, Ni, Cu, Zn, Cd, Pb, Mn, and As) in 10 desert plants and their growth matrices. In addition, the single factor pollution index and Nemerow comprehensive pollution index were used to evaluate soil pollution. Based on the biological accumulation factor and the biological concentration factor, the fuzzy evaluation method was used to evaluate plant extraction/stability potentials for heavy metals. The results showed that Cd, Cu, Ni, and Cr in the soil around the tailings pond were at the heavy pollution level, Mn and Pb pollution was moderate, and Zn and As pollution was light. The matrix factorization model showed that Cu and Ni came from industrial pollution; Cd and Cr came from atmospheric deposition and agricultural pollution; Pb came from traffic pollution; and Mn, Zn, and As came from natural sources. The metal contents of the desert plants exceeded the standard for normal plants, of which Cr contents in the *Atriplex patens* and *Ammophila breviligulata* Fernald aboveground parts were 35.63 and 53.77 mg/kg respectively, and the Ni contents in the *Klasea centauroides* subsp. *polycephala* (Iljin) L. Martins underground parts and the *A. breviligulata* Fernald aboveground parts were 102.67 and 101.67 mg/kg, respectively, exceeding the maximum toxicity threshold for Cr and Ni. *Ammophila breviligulata* Fernald had the highest plant comprehensive extraction coefficient (CEI) and plant comprehensive stability coefficient (CSI) at 0.81 and ~~0.83 respectively,~~ indicating that it has strong heavy metal extraction and stabilization abilities. Therefore, *A. breviligulata* Fernald can be selected as a remediation species for heavy metal pollution in the soil around tailings reservoir areas.

Keywords: Arid region; Tailings pond; Heavy metal pollution; Fuzzy evaluation; Desert plants; Phytoremediation

1. Introduction

Soil heavy metal pollution has gradually become a global problem due to increased industrialization and urbanization (Hanfi et al., 2020; Jeong et al., 2020; Khodaverdiloo et al., 2020; Yu et al., 2020). In particular, mining causes damage to regional vegetation (Li et al., 2020; Mwitwa et al., 2012) and its tailings from ore refining are an important source of environmental pollution

(Consuelo et al., 2021; Zhang et al., 2021). Tailings are residual wastes from the processing and production of ores and industrial minerals. In particular, they contain unstable primary and secondary minerals (Huang et al., 2012). It is estimated that more than 10 billion tons of tailings are produced in the world every year and a large amount of tailings accumulate leading to a large environmental footprint in time and space (Adiansyah et al., 2015). Generally, tailings are acidic (Mendez et al., 2008), mainly composed of silt or sand sized particles, lack nutrient elements (N, P, K) to support plant growth, and contain almost no organic matter, but their heavy metal contents are high (Ginocchio, 2000; Mendez and Maier, 2008). In arid climates, the fine particles on the tailings surface are rich in pollutants and are vulnerable to the influence of wind-induced dispersion and hydraulic action. This means that they continue to diffuse to the surrounding environment, eventually leading to expansion of the polluted area (G et al., 2015; Mendez et al., 2008). In addition, the large evaporation rate in arid areas makes the water rapidly migrate upward through capillary action, providing supersaturation conditions for the formation of the secondary evaporation solid phase by weathering (Khorasanipour, 2015). Therefore, soil heavy metal pollution in arid areas may pose a greater threat to the ecological environment than in other areas, such as humid or semi-humid areas (Punia, 2021).

Heavy metal elements are not affected by biodegradation, but are converted between various species, which means that it is very difficult to remediate heavy metals in soil (Sarwar et al., 2017). In recent decades, researchers have tried to develop effective remediation technologies for contaminated soils (Khodaverdiloo et al., 2020). Currently, three general methods are used to remediate heavy metal pollution in soil. These are physical remediation, chemical remediation, and biological remediation (Roychowdhury et al., 2018). Physical remediation methods include soil replacement, soil washing, and vitrification, while soil chemical methods include stabilization and extraction (Ashraf et al., 2019; Roychowdhury et al., 2018). However, physical and chemical remediation methods are not suitable for large-scale application due to high cost, low efficiency, or possible damage to the soil structure (Khodaverdiloo et al., 2020; Zhao et al., 2014). In contrast, phytoremediation methods (bioremediation methods) have attracted extensive attention due to their advantages, such as being solar energy driven, their cost-effectiveness, and they are environmentally friendly (Sarwar et al., 2017). Therefore, evaluating the phytoremediation potential for heavy metals in heavy metal contaminated sites in arid areas is of great significance when attempting to enrich the database of heavy metal enriched plant resources and promote the wide applicability of heavy metal phytoremediation technology.

Phytoremediation technology refers to the various technologies that use natural plants or transgenic plants to repair polluted environments (Flathman et al., 1998). Phytoremediation techniques include plant extraction, plant stabilization, rhizosphere filtration, plant degradation, and plant volatilization (Karaca et al., 2018), while phytoextraction and plant stabilization techniques are usually used to remediate heavy metal pollution in soil (Mendez et al., 2008; Wong, 2003). Plant extraction absorbs and transfers heavy metal pollutants to the aboveground part through plant roots and finally removes the heavy metals in soil through artificial harvesting and treatment (Lam et al., 2018; Padmavathiamma et al., 2007). Plants also stably absorb and accumulate heavy metals in soil through their roots, which reduces heavy metal mobility and effectiveness and stabilizes (harmlessness) heavy metals in soil (Lam et al., 2018; Padmavathiamma et al., 2007). Based on these effects, this study evaluated the main role of phytoremediation technology in soil heavy metal remediation by using fuzzy theory and the plant comprehensive extraction coefficient (CEI) and

plant comprehensive stability coefficient (CSI). The CEI and CSI for plants can reflect the removal and stabilization effect of plants on different heavy metals in soil and provide a more reasonable basis that can be used to select plants for the remediation of complex heavy metal contaminated sites.

In summary, the aims of this study were (1) to explore the impact of tailings accumulation on the enrichment of heavy metals in the surrounding soil, (2) clarify the sources of heavy metals in soil and provide reasonable suggestions for the effective prevention and control of heavy metal pollution in soil, and (3) evaluate the phytoremediation potential of native plants to provide a reference basis for the phytoremediation of heavy metal contaminated soil.

2. Materials and methods

2.1. Study area description and sample collection

The copper-nickel mine tailings pond was built in 1999 and is located in northern Xinjiang Province and in the eastern part of Fuyun County (Fig. 1). The design level of the pond capacity is grade Ⅴ and the floor area is about 1.5×10^5 m^2. The slag waste is disposed of by the traditional wet discharge method and the discharge volume is about 1,000 t/d. The altitude of the area ranges from 500–900 m and the soil types are mainly light brown calcium soil and typical brown calcium soil. The area is subjected to a continental cold temperate climate. It is dry and windy in spring, but cold in winter. There are also large temperature differences between day and night. The annual average temperature is 3.0℃, the annual precipitation is 186.4 mm, the annual evaporation is 1,829.7 mm, the annual extreme maximum temperature is 42.2℃, and the extreme minimum temperature is -51.5℃. It is one of the high, cold regions in China. In September, 2021, the natural plants around the tailings pond were investigated and sampled. During this survey, 10 native plants belonging to eight families were collected and recorded (Table 1). The plant samples were then divided into underground and aboveground parts and each plant was replicated three times. Soil samples from around the plant roots were collected at the same time as the plant samples. The soil sampling depth was 0–20 cm and a total of 60 plant samples and 30 soil samples were collected.

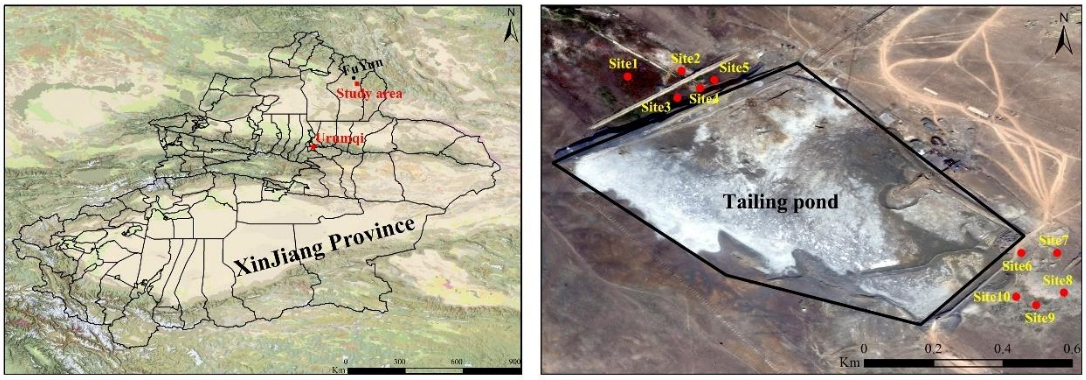

Figure 1. Location of the study area and distribution of the sample points.

Table 1

Composition of native plant species around the tailing compounds.

Species	Site	Family	Life form
Polygonum aviculare L.	9	Polygonaceae	Annual grass
Salsola ruthenica	6	Chenopodiaceae	
Halogeton arachnoideus Moq.	10	Amaranthaceae	
Atriplex patens	8	Chenopodiaceae	
*Klasea centauroide*s subsp. *polycephala* (Iljin) L. Martins	4	Compositae	Perennial grass
Limonium sinense (Girard) Kuntze	1	Plumbaginaceae	
Sphaerophysa salsula (Pall.) DC.	2	Leguminosae	
Ammophila breviligulata Fernald	5	Gramineae	
Phragmites australis (Cav.) Trin. ex Steud	3	Gramineae	
Peganum harmala L.	7	Zygophyllaceae	

2.2. Sample processing and determination

The plant samples were first washed with tap water and then three times with deionized water. They were placed in an oven at 105℃ for 20 minutes, dried at 70℃ to a constant weight, ground with a grinder to pass through a 100 mesh nylon sieve, and bagged. Then, 0.2 g subsamples of the plants were weighed out, placed in a microwave digester, and digested in an HNO_3-H_2O_2 digestion system (HNO_3:H_2O_2 = 5:1, volume ratio) until the liquid clarified. The soil samples were dried after removing impurities, such as stones and animal and plant residues. These samples were then ground with agate mortar through a 200 mesh nylon sieve, and bagged until needed. Then, 0.1 g subsamples of the soil were digested in a microwave digester under a HNO_3-HF-$HClO_4$ digestion system (HNO_3:HF:$HClO_4$ = 3:1:1, volume ratio) until the liquid clarified. The experiment was verified using the blank control method, the double parallel sample method, and the standard addition recovery method to ensure the accuracy of the experimental and determination processes. The supernatants from the subsamples were removed and the contents of eight heavy metals (Cr, Ni, Cu, Zn, Cd, Pb, Mn, As) were determined by inductively coupled plasma mass spectrometry (ICP-MS).

2.3. Evaluation of heavy metal pollution in soil

2.3.1. Single factor pollution index method

The single factor pollution index (P_i) is a common method used to evaluate the degree to which a soil has been polluted with heavy metals (Wang et al., 2011). The index is used to evaluate environmental quality by comparing the measured value with the standard value. The calculation formula is as follows (Wang et al., 2011; Young et al., 2021):

$$P_i = C_i / S_i \tag{1}$$

where P_i is the single-component contamination index, C_i is the measured concentration of examined metal *i* in the soil, and S_i is the background concentration of metal *i*. This study took the background value for heavy metals in Xinjiang soil as the standard value. The evaluation results were divided into five grades: $P_i \leq 0.7$, safe; $0.7 < P_i \leq 1.0$, warning; $1 < P_i \leq 2$, slight pollution; $2 < P_i \leq 3$, moderate pollution; and $P_i > 3$, heavy pollution.

2.3.2. Nemerow comprehensive pollution index method

The Nemerow comprehensive pollution index is used to calculate the comprehensive pollution

effects of all assessed heavy metals. It can comprehensively reflect the effects of various heavy metals on soil and avoids the weakening of heavy metal weights caused by averaging. The calculation formula is as follows (Liang et al., 2011; Liu et al., 2020; Nemerow, 1991):

$$P_{com} = \sqrt{\frac{P_{max}^2 + P_{ave}^2}{2}} \quad (2)$$

where P_{com} is the composite contamination index, P_{ave} is the average value of the single-factor index, and P_{max} is the maximum value of the single-factor index. The evaluation results were divided into five grades: $P_{com} \leq 0.7$, safe; $0.7 < P_{com} \leq 1.0$, warning; $1 < P_{com} \leq 2$, light pollution; $2 < P_{com} \leq 3$, moderate pollution; and $P_{com} > 3$, heavy pollution.

2.4. Evaluation of phytoremediation potential

2.4.1. Enrichment characteristics of heavy metals in plants

Heavy metals absorbed by plants are mainly characterized by the biological accumulation factor (BAF) and the biological concentration factor (BCF). Their calculation formulas are as follows (Elalaoui et al., 2021):

$$BAF_i = C_{shoot}^i / C_{soil}^i \quad (3)$$

$$BCF_i = C_{root}^i / C_{soil}^i \quad (4)$$

where i represents the ith heavy metal element; BAF_i is the enrichment coefficient of plants for the ith heavy metal, which is a parameter that is used to evaluate the ability of plant stems and leaves to extract heavy metals from soil; BCF_i is the stability coefficient of plants for the ith heavy metal and is a parameter used to evaluate the ability of plant roots to stabilize heavy metals; C_{shoot}^i is the content of the ith heavy metal in the aboveground part of the plant; C_{root}^i is the content of the ith heavy metal in the underground part of the plant; and C_{soil}^i is the content of the ith heavy metal in the plant growth matrix.

2.4.2. Comprehensive extraction index for plants

The comprehensive extraction index (CEI) for plants is based on fuzzy synthesis and can be used to evaluate the plant comprehensive extraction potential for various heavy metals under multiple heavy metal combined pollution conditions. The calculation formula is as follows (Hołtra et al., 2020; Zhao et al., 2014):

$$CEI = \left(\frac{1}{n}\right)\sum_{1}^{n} u_i \quad (5)$$

$$u_i = \begin{cases} 0 & BAF = BAF_{min} \\ \dfrac{BAF - BAF_{min}}{BAF_{max} - BAF_{min}} , & BAF_{min} < BAF < BAF_{max} \\ 1 & BAF = BAF_{max} \end{cases} \quad (6)$$

where CEI is the comprehensive extraction index for plants; n is the total number of heavy metals analyzed; BAF is the biological accumulation factor of plants for the ith heavy metal; BAF_{max} and BAF_{min} are the maximum and minimum values of the biological accumulation factor for ith

heavy metals in the investigated plants, respectively; and u_i is the fuzzy membership value. The comprehensive extraction potential of plants is divided into three grades, poor ($\mathrm{CEI} \leq 0.4$), good ($0.4 < \mathrm{CEI} < 0.7$), and excellent ($\mathrm{CEI} \geq 0.7$).

2.4.3. Plant comprehensive stability index

The plant comprehensive stability index (CSI) is based on fuzzy synthesis and can be used to evaluate the plant root comprehensive stability potential for various heavy metals under multiple heavy metal combined pollution conditions. The calculation formula is as follows:

$$CSI = \left(\frac{1}{n}\right)\sum_{1}^{n} u_i \tag{7}$$

$$u_i = \begin{cases} 0 & BCF = BCF_{min} \\ \dfrac{BCF - BCF_{min}}{BCF_{max} - BCF_{min}} \quad , & BCF_{min} < BCF < BCF_{max} \\ 1 & BCF = BCF_{max} \end{cases} \tag{8}$$

where CSI is the comprehensive stability index for a plant; n is the total number of heavy metals analyzed; BCF is the biological accumulation factor of plants for the ith heavy metal; BCF_{max} and BCF_{min} are the maximum and minimum values of the biological concentration factor for ith heavy metals in the investigated, plants, respectively; and u_i is the fuzzy membership value. The comprehensive stability potential of plants is divided into three grades, poor ($\mathrm{CEI} \leq 0.4$), good ($0.4 < \mathrm{CEI} < 0.7$), and excellent ($\mathrm{CEI} \geq 0.7$).

2.5. Data processing and analysis

IBM SPSS statistics 22.0 (IBM Corp., Armonk, NY, USA) was used to statistically analyze the heavy metal contents in the soil, EPA PMF 5.0 was used for the source analysis of heavy metals in soil, and Origin 2020 (OriginLab Corporation, Northampton, MA, USA) and ArcGIS 10.6 (ESRI Inc., Redlands, CA, USA) were used for the graphics.

[illegible] Results and analysis

3.1. Statistical characteristics of soil heavy metal content

According to the descriptive statistical analysis (Table 2), the Cr, Ni, Cu, Zn, Cd, Pb, Mn, and As contents in the soil samples were 255.2–911.3, 393.6–2861.1, 307.9–1654.3, 99.4–151.6, 2.7–12.7, 18.1–69.6, 1318.5–1810.8, and 5.4–4.9 mg/kg, with average contents of 529.0, 1168.0, 738.5, 118.2, 6.4, 40.1, 1600.3, and 13.9 mg/kg, respectively. The average Cr, Ni, Cu, Zn, Cd, Pb, Mn, and As contents in the soil samples were 10.7, 43.9, 27.7, 1.7, 53.3, 2.1, 2.3, and 1.2 times more than the background values for heavy metals in Xinjiang soils, respectively. Compared to the national soil environment class II standard, Cr, Ni, Cu, Cd, and As in the soil samples exceeded the standard by about 3.5, 29.2, 14.8, and 21.3 times, respectively. The variation indexes for Ni and Cu in the soil were 61.72% and 53.16% respectively, indicating that Ni and Cu are causing serious localized pollution. The variation indexes for Cr, Cd, Pb, and As were 47.19%, 37.94%, 43.25%, and 38.22%, respectively, which showed that the variation indexes were relatively weak. The variation indexes

for Zn and Mn were 11.87% and 9.38%, respectively, which are less than 25% and shows that the variation was low and that they are less affected by external conditions.

Table 2
Statistics for heavy metal content in the soil around the tailing reservoir area (n = 30)

Heavy metal	Cr	Ni	Cu	Zn	Cd	Pb	Mn	As
Mean(mg/kg)	529.0	1168.0	738.5	118.2	6.4	40.1	1600.3	13.9
Minimum(mg/kg)	255.2	393.6	307.9	99.4	2.7	18.1	1318.5	5.4
Maximum(mg/kg)	911.3	2861.1	1654.3	151.6	12.7	69.6	1810.8	24.9
Coefficient of Variation (%)	47.19	61.72	53.16	11.87	37.94	43.25	9.38	38.22
BG_1[a] (mg/kg)	49.3	26.6	26.7	68.8	0.12	19.4	688	11.2
BG_2[b] (mg/kg)	150	40	50	200	0.3	250	-	40

[a]BG_1: background values for heavy metals in Xinjiang soil
[b]BG_2: National Soil Environment class II standard

3.2. Evaluation of heavy metal pollution in soil

The pollution caused by different heavy metal elements in the soil was evaluated using Formula (1). The results (Fig. 2) show that the single factor pollution indexes for heavy metal elements Cd, Cu, Ni, Cr, Mn, Pb, Zn, and As were between 22.5–108.5, 11.5–62.0, 14.8–107.6, 1.7–6.1, 1.9–2.6, 0.9–3.6, 1.4–2.2, and 0.5–2.2, respectively. Among them, the average single factor pollution indexes for Cd, Cu, Ni, and Cr were 53.2, 27.7, 43.9, and 3.5, respectively, which were greater than 3 and meant that they reached the heavy pollution grade; the single factor pollution index mean values for Mn and Pb were 2.3 and 2.1, respectively, which were at the moderate pollution level; and the single factor pollution index mean values for Zn and As were 1.7 and 1.2, respectively, which were at the light pollution level. The Nemerow comprehensive pollution index results, calculated using Formula (2) (Fig. 2), showed that the Nemerow pollution indexes for all sample points ranged from 25.0 to 79.0, with an average of 43.9, which was at the heavy pollution level. In general, the soil was heavily polluted with metals and remediation measures were urgently needed.

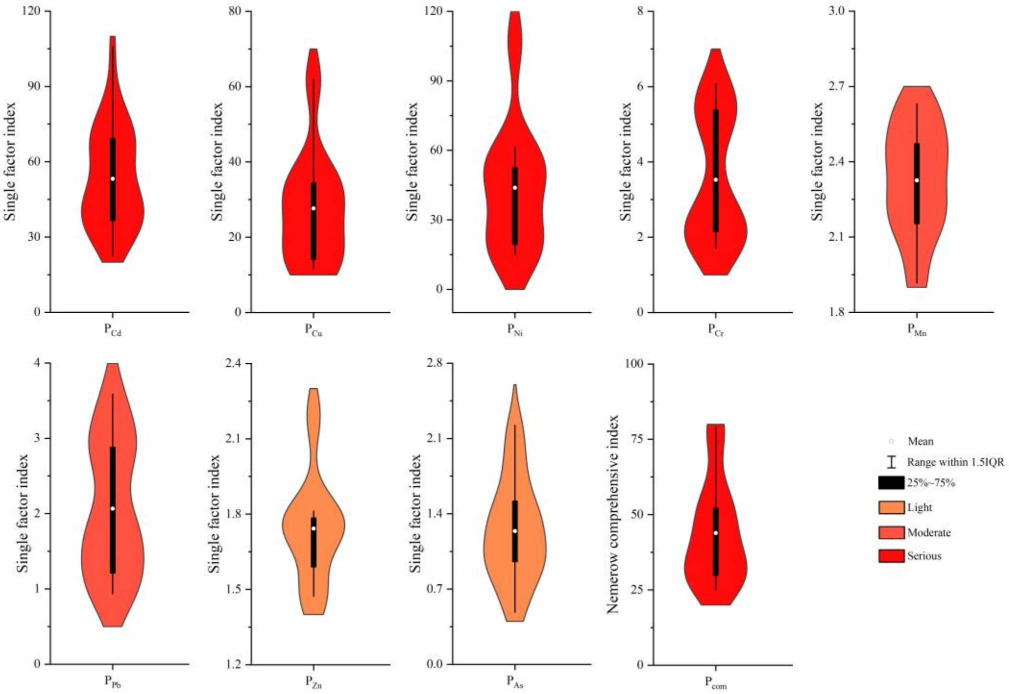

Figure 2. Descriptive statistics for the heavy metal pollution indexes.

3.3. Source analysis of heavy metals in soil

Correlation analyses and the positive matrix factorization (PMF) model can be used to analyze the sources of heavy metals in soil (Huang et al., 2021). The Pearson correlation analysis of the heavy metal contents in the soils showed that there were correlations among most of the heavy metals (Fig. 3). Among them, the correlations between Ni and Cu, Zn, Pb, Cr and Cd, Pb, Cu, and Zn, and Pb, Cd, and Pb reached a very significant level ($P < 0.01$). These results showed that there were strong correlations among some of the heavy metals. The correlations between Ni and Cd, and Cu and Cd, Zn, and Pb reached significant levels ($P < 0.05$). The analysis results for the sources of heavy metals in the soil obtained from the study area using the PMF model (Fig. 3) show that Factor 1 contributed most to Cd and Cr levels at 44.6% and 42.8%, respectively. Atmospheric deposition is an important source of Cr and Cd in soil. The annual input of Cd and Cr into soil through atmospheric deposition is 493 and 7392 t/a, respectively (Luo et al., 2009). In addition, Cd is also considered to be a symbol element of agricultural activities because chemical fertilizers, livestock manure, and irrigation water are important sources of Cd (Cai et al., 2019; Pan et al., 2016). It has also been reported that the Cd inputs due to fertilizer, livestock manure, and irrigation water into agricultural soil in China are 113, 778, and 30 t/a, respectively (Luo et al., 2009). The study area was located in the arid area of Northwest China, which uses large amounts of water for agricultural irrigation and has developed animal husbandry. These activities may have led to the Cd enrichment seen in the study soil. Therefore, Factor 1 came from composite pollution sources, including atmospheric deposition and agricultural pollution sources. Factor 2 contributed most to Cu and Ni levels at 62.5% and 66.5%, respectively. The correlation analysis showed that the correlation

between Cu and Ni was high at 0.88, indicating that Cu and Ni had the same pollution source. The sampling points were located near the copper nickel mining, smelting, and tailings pond, which suggests that Factor 2 is based on industrial pollution sources. Factor 3 contributed 54.5%, 47.9%, and 40.0% to Mn, As, and Zn, respectively. Table 1 shows that the variation indexes for Mn, As, and Zn were low; and the single factor pollution evaluation results (Fig. 2) showed that Mn was moderately polluting, whereas As and Zn were slightly polluting. These results consistently showed that Mn, As, and Zn were less disturbed by human factors. Previous studies have shown that Mn mainly came from soil parent materials (Luo et al., 2022; Lv, 2019; Lv et al., 2015). Therefore, Factor 3 was based on natural sources. Factor 4 made the greatest contribution toward Pb levels at 41.2%. Lead is the main marker for traffic emissions caused by fuel combustion, and engine and catalyst use (Guan et al., 2018). It is estimated that automobile exhaust emissions account for about two-thirds of global lead emissions (Cui et al., 2018; Fei et al., 2020). Therefore, Factor 4 was based on traffic pollution sources.

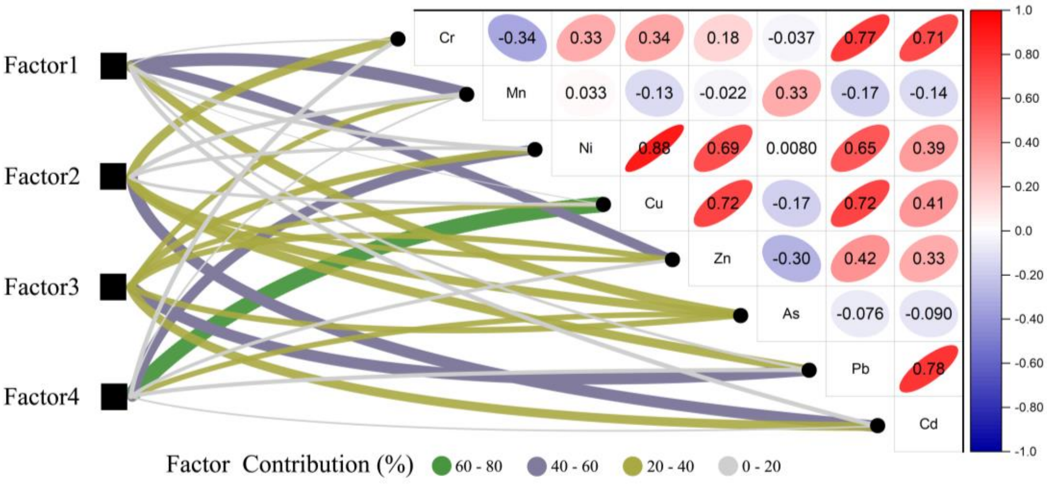

Figure 3. Correlation analysis and source analysis of the heavy metals.

3.4. Heavy metal content in plants

The heavy gold contents in different plants around the tailings pond are shown in Fig. 4. It showed that the heavy metal levels in different plants or different parts of the same plant showed large variations, which may be closely related to the physical and chemical properties of the soil, the occurrence forms of heavy metals, and the characteristics of the plants themselves. The contents of eight heavy metals (Cr, Ni, Cu, Zn, Cd, Pb, Mn, and As) in the aboveground parts of 10 plants varied from 2.02–53.77, 24.57–178.67, 7.75–101.67, 10.22–77.37, 6.19–47.13, 0.12–1.72, 0.01–0.94, and 0.28–2.91 mg /kg, respectively, and the Cr, Ni, Cu, Zn, Cd, Pb, Mn, and As variation ranges for their contents in the underground parts of the 10 plants were 5.34–21.50, 26.10–255.33, 21.0–-102.67, 10.22–91.10, 10.7–65.73, 0.17–0.32, 0.03–1.16, and 0.42–5.41 mg/kg, respectively. In general, the Ni, Cu, Pb, and As contents in the underground parts of most plants were higher than those in the aboveground part, while Cr, Mn, and Cd were lower than those in the aboveground parts. The heavy metal contents in plants from high to low were Mn > Ni > Cu > Zn > Cr > Pb > As > Cd. Compared to the maximum values for normal heavy metal contents in plants (Kabata et

al., 2007), the heavy metal elements Ni (Ni normal range: 0.1–5 mg/kg) and Cr (Cr normal range: 0.1–0.5 mg/kg) in the underground or aboveground parts of all plants exceeded the maximum value for the normal content range. The Cr content in the aboveground parts of *Atriplex patens* and *Ammophila breviligulata* exceeded the maximum toxicity threshold (toxicity threshold range for Cr: 5–0.5 mg/kg). The Ni content in the underground part of *Klasea centauroides* and the aboveground part of *A. breviligulata* also exceeded the maximum toxicity threshold range (toxicity threshold range for Ni: 10–100 mg/kg). Except *P. australis* and *L. sinense*, the Cu levels in the underground parts of other plants exceeded the maximum normal level (normal range for Cu: 5–30 mg/kg), while the Cu levels in most aboveground parts were within the normal range. The Cd levels in the plants (aboveground or underground parts) exceeded the maximum for the normal range (normal range for Cd: 0.01–0.2 mg/kg), except for *L. sinense*, *S. salsula* and *P. australis*. The As content in all plants (aboveground and underground parts), except for *A. breviligulata*, did not exceed the maximum normal range (normal range for As: 1–1.5 mg/kg), and the Mn, Zn, and Pb contents in all plants (aboveground and underground parts) were within the normal range (normal range for Mn: 30–300 mg/kg, normal range for Zn: 25–250 mg/kg, and normal range for Pb: 5–10 mg/kg).

[illegible] *Heavy metal absorption characteristics of plants*

The biological accumulation factor (BAF) shows the ability of plant stems and leaves to accumulate heavy metals, which means that it reflects the ability of plants to remove heavy metals from soil. Table 3 shows that there were obvious differences in the accumulation capacity of plants for heavy metals. It also shows the differences among different plants for the same element and in the same plant for different elements. The minimum biological accumulation factors for the 10 plants around the tailings pond for Cr, Mn, Ni, Cu, Zn, As, Cd, and Pb are 0.006, 0.003, 0.014, 0.051, 0.010, 0.001, and 0.008, respectively, and the maximum values are 0.110, 0.113, 0.104, 0.107, 0.457, 0.133, 0.190, and 0.074, respectively. Among the 10 plants, *A. patens* has a strong ability to extract Cr, *P. aviculare* has a strong ability to extract Zn, *S. ruthenica* has a strong ability to extract Cd, and *A. breviligulata* shows a strong extraction ability for Mn, Ni, Cu, As, and Pb.

Plant stabilization is the use of plant roots to absorb and accumulate toxic metals in soil, thereby reducing the bioavailability and migration of heavy metals in soil (Flathman et al., 1998). The biological concentration factor (BCF) can reflect the ability of plants to stabilize heavy metals in soil. Table 4 shows that the minimum plant stability coefficients for the 10 plants around the tailings pond for Cr, Mn, Ni, Cu, Zn, As, Cd, and Pb were 0.007, 0.019, 0.007, 0.022, 0.105, 0.008, 0.004, and 0.007, respectively, and the maximum values were 0.066, 0.162, 0.143, 0.127, 0.553, 0.567, 0.154, and 0.138, respectively. Among them, *S. ruthenica* can strongly stabilize Cr, *P. harmala* can strong stabilize Ni, Cu, and Cd, and *A. breviligulata* can strongly stabilize Mn, Zn, As, and Pb.

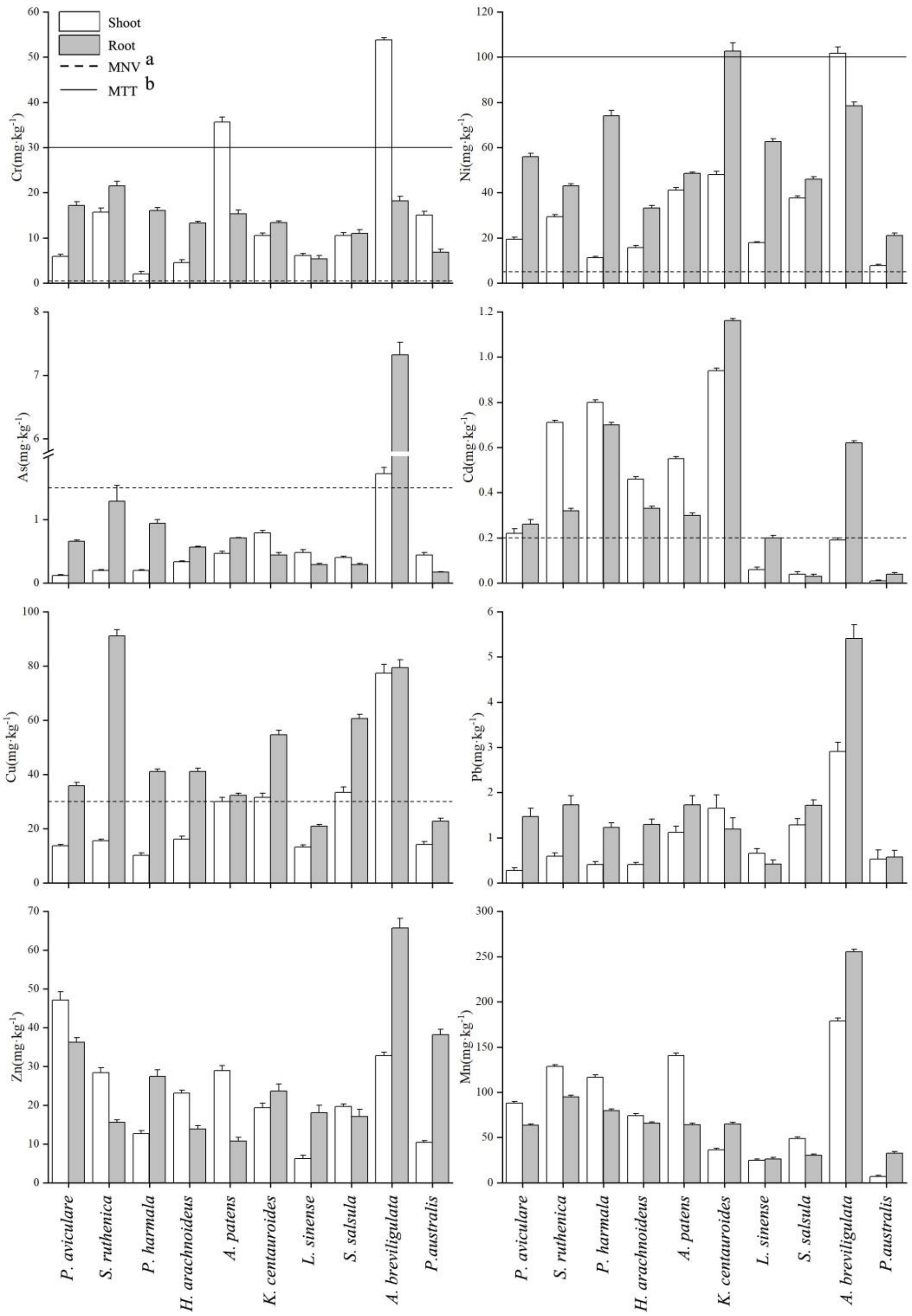

Figure 4. Heavy metal contents in the aboveground and underground parts of 10 desert plants (a: MNV, Maximum normal value, b: MTT, Maximum toxicity threshold).

Table 3

Biological accumulation factors (BAFs) for desert plants around the tailings pond

Species	BAF							
	Cr	Mn	Ni	Cu	Zn	As	Cd	Pb
P. aviculare	0.012	0.049	0.014	0.023	0.457	0.01	0.05	0.008
S. ruthenica	0.048	0.087	0.074	0.017	0.238	0.019	0.19	0.032
P. harmala	0.006	0.08	0.022	0.032	0.084	0.035	0.176	0.02
H. arachnoideus	0.015	0.044	0.022	0.042	0.198	0.039	0.083	0.017
A. patens	0.11	0.078	0.04	0.054	0.284	0.042	0.109	0.031
K. centauroides	0.014	0.023	0.03	0.039	0.158	0.049	0.097	0.029
L. sinense	0.008	0.019	0.013	0.016	0.051	0.031	0.007	0.011
S. salsula	0.012	0.031	0.027	0.02	0.161	0.027	0.005	0.019
A. breviligulata	0.106	0.113	0.104	0.107	0.276	0.133	0.03	0.074
P. australis	0.018	0.017	0.003	0.014	0.084	0.022	0.001	0.01

Table 4

Biological concentration factors (BCFs) for desert plants around the tailings pond

Species	BCF							
	Cr	Mn	Ni	Cu	Zn	As	Cd	Pb
P. aviculare	0.036	0.035	0.04	0.06	0.351	0.056	0.059	0.043
S. ruthenica	0.066	0.064	0.109	0.099	0.131	0.121	0.086	0.094
P. harmala	0.048	0.055	0.143	0.127	0.182	0.164	0.154	0.061
H. arachnoideus	0.045	0.039	0.047	0.108	0.118	0.065	0.06	0.055
A. patens	0.047	0.036	0.047	0.059	0.105	0.064	0.06	0.047
K. centauroides	0.019	0.042	0.063	0.067	0.193	0.027	0.119	0.021
L. sinense	0.007	0.02	0.046	0.026	0.15	0.018	0.022	0.007
S. salsula	0.012	0.019	0.033	0.037	0.141	0.02	0.004	0.025
A. breviligulata	0.036	0.162	0.081	0.109	0.553	0.567	0.097	0.138
P. australis	0.008	0.019	0.007	0.022	0.309	0.008	0.005	0.011

3.6. Comprehensive evaluation of phytoremediation potential

The plant comprehensive extraction index and plant comprehensive stability index results, calculated based on Fuzzy evaluation (Fig. 5), showed that among the 10 plants investigated, the plant comprehensive extraction indexes for *P. australis*, *L. sinense*, *S. salsula*, *H. arachnoideus*, *K. centauroide*s, *P. harmala*, and *S. ruthenica* were 0.04, 0.05, 0.14, 0.23, 0.25, 0.26, and 0.30, respectively, which were less than 0.4, which meant that their comprehensive removal potentials for heavy metals were poor. The plant comprehensive extraction index for *A. patens* was 0.52, which meant that its comprehensive removal potential for heavy metals was good; and the plant comprehensive extraction index for *A. breviligulata* was 0.83, which means that its comprehensive removal potential level for heavy metals was excellent, indicating that it had a strong comprehensive removal ability for heavy metals. The plant comprehensive stability indexes for *P. australis*, *L. sinense*, *S. salsula*, *A. patens*, *K. centauroide*s, *P. aviculare*, and *H. arachnoideus* were smaller at 0.07, 0.07, 0.08, 0.28, 0.29, 0.31, and 0.34, respectively, which are all less than 0.4. Therefore, their potential stability indexes for heavy metals are poor, indicating that their comprehensive ability to stabilize heavy metals is weak. The plant comprehensive stability indexes for *S. ruthenica* and *P. harmala* were 0.53 and 0.60 respectively, which meant that their potential stability grades for heavy metals were good. The plant comprehensive stability index for *A. breviligulata* was 0.81, which meant that its stability potential index for heavy metals was excellent, indicating that it had strong comprehensive ability to stabilize heavy metals. Therefore, *A. breviligulata* had greater remediation potential for heavy metals in soil than the other plants.

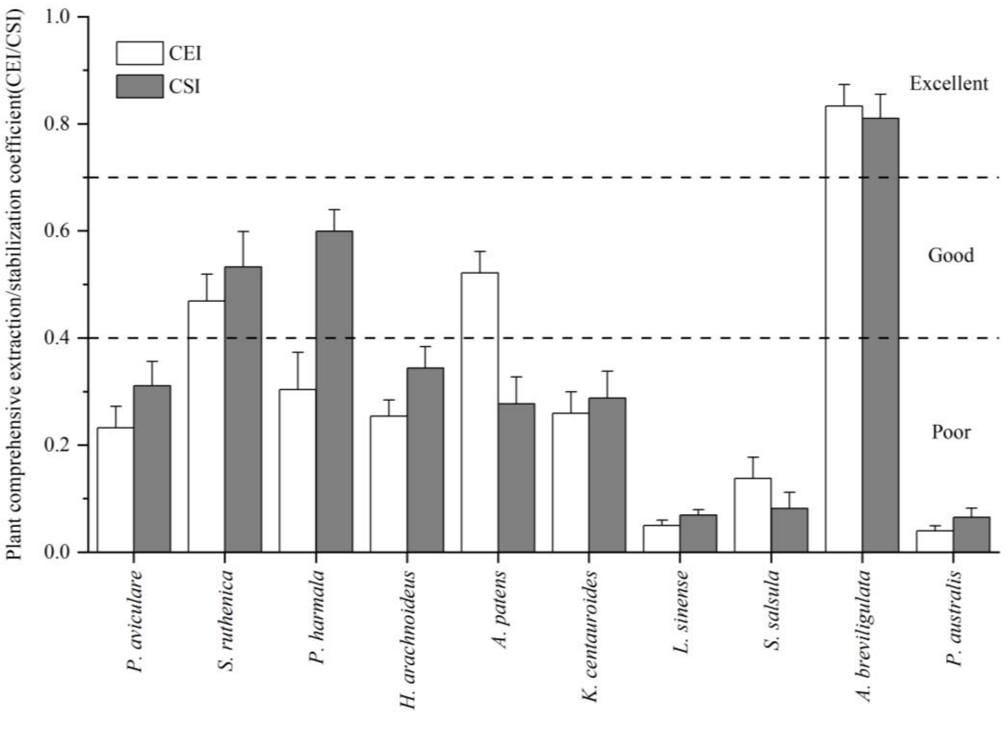

Figure 5. Plant comprehensive enrichment/stability coefficients for the 10 desert plants.

4. Discussion

Tailings ponds are one of the important sources of soil heavy metal pollution. Climatic factors (temperature, precipitation, and wind) play an important role in aggravating the migration and diffusion of heavy metal pollution (Meza Figueroa et al., 2009b; Punia, 2021). Temperature affects the oxidation-reduction reaction rate in the environment and the oxidation-reduction reaction affects the mobility of heavy metals. Previous research has shown that, compared to the initial stable heavy metal activities, the oxidation-reduction reaction in mine tailings led to an increase in Pb, Zn, and Mn activities by 42–69% (Trifi et al., 2019). In cold regions, the amounts of heavy metals leached in tailings increases with the increase in temperature (Fu et al., 2019). Kalinovic et al. (2019) investigated the heavy metal contents in soil and found that wind direction had an important impact on the distribution of heavy metal pollution in soil and that the heavy metal contents in the soils that were in the path of the prevailing wind were higher. Tailings have low pHs, water retention capacities, and cation exchange capacities, which means that heavy metals in tailings exhibit strong fluidity (Meza-Figueroa et al., 2009a) and that heavy metals will show accelerated diffusion under high precipitation and snow melt conditions. In arid areas, strong surface evaporation promotes the formation of sulfates on the surface of mining wastes (Khorasanipour et al., 2018) These sulfates are prone to aeolian diffusion and have a negative impact on the surrounding environment (Meza-Figueroa et al., 2009b). The study area was located in the arid area of Northwest China where the temperature difference between day and night is large, it is hot in summer and cold in winter, and there is strong evaporation and erosion (wind erosion, hydraulic erosion, and freeze-thaw erosion). Therefore, there may be a potential risk of heavy metal diffusion in the tailings pond. The evaluation results for heavy metals in the soil around the copper-nickel tailings pond in this study showed that Cd, Cu, Ni, and Cr were at the heavy pollution level, Mn and Pb were at the moderate pollution level, and Zn and As were at the light pollution level. The source analysis using the PMF model showed that pollution by Cu, Ni, Cr, and Cd was closely related to industrial activities, such as mining, ore smelting, and tailings accumulation. Li et al. (2022) found that there were many kinds of heavy metal pollution in the soil around a copper nickel mine tailings pond, among which Cu, Ni, and Cr were the main environmental pollutants derived from tailings. Their results were similar to the results obtained from this study. Liang et al. (2017) collected water, soil, rice, and vegetable samples in an area near a tailings pond and found that these samples were contaminated with heavy metals and posed potential health risks to nearby residents. Therefore, the diffusion of heavy metals in tailings ponds will cause serious pollution to the environment and pose a serious threat to human life and health.

Phytoremediation uses the accumulation of elements by plant stems, leaves, and roots to remove and stabilize heavy metals in soil (Fig. 6). Furthermore, the plant canopy formed after phytoremediation can reduce the near surface wind speed and the diffusion of fine particle pollutants, while the underground root network can prevent rainfall erosion and leaching, and provide a suitable rhizosphere environment for heavy metal precipitation (Cameselle et al., 2013; Houben et al., 2013). In general, identifying the natural vegetation growing on contaminated sites and the selection of metal tolerant plants with potential remediation value are some of the most important components of a localized phytoremediation strategy (Matanzas et al., 2021). Mousavi et al. (2020) evaluated the remediation potential of plant species growing in a heavy metal polluted saline alkali soil and found that seven plants (*L. arborescens*, *A. santolina*, *P. gnaphalodes*, *Z. eurypteru*, *P. harmala*, *P.*

olivieri, and *A. javanica*) have good heavy metal stabilization effects, while *Z. eurypterum* and *A. javanica* also have good heavy metal and relatively high heavy metal extraction abilities, respectively. Liu et al. (2016) evaluated the plant stability and plant extraction potential of desert plants naturally growing around tailings ponds in Altay, Xinjiang Province, China. The evaluation results showed that *S. schmidt* was most suitable for stabilizing Cu, *K. caspica* was most suitable for stabilizing Cd, and *P. Aviculare* had a high extraction potential for Cu. Zhao et al. (2014) used a fuzzy comprehensive evaluation to evaluate the comprehensive heavy metal extraction potential of woody plants growing on heavy metal contaminated sites and found that *B. papyrifera* could extract many kinds of heavy metals at the same time. In terms of extraction potentials for single heavy metals, this study found that *A. patens* had a strong ability to extract Cr; *P. aviculare* had a strong Zn extraction ability; *S. ruthenica* had a strong Cd extraction ability; *A. breviligulata* had strong Mn, Ni, Cu, As, and Pb extraction abilities. In terms of single heavy metal extraction and stabilization abilities, this study found that *S. ruthenica* had a strong ability to stabilize Cr; *P. harmala* had a strong ability to stabilize Ni, Cu, and Cd; and *A. breviligulata* had a strong ability to stabilize Mn, Zn, As, and Pb. The evaluation results for plant heavy metal comprehensive extraction/stabilization potential based on fuzzy mathematics showed that the plant comprehensive extraction and plant comprehensive stability indexes for *A. breviligulata* were the highest, indicating that *A. breviligulata*

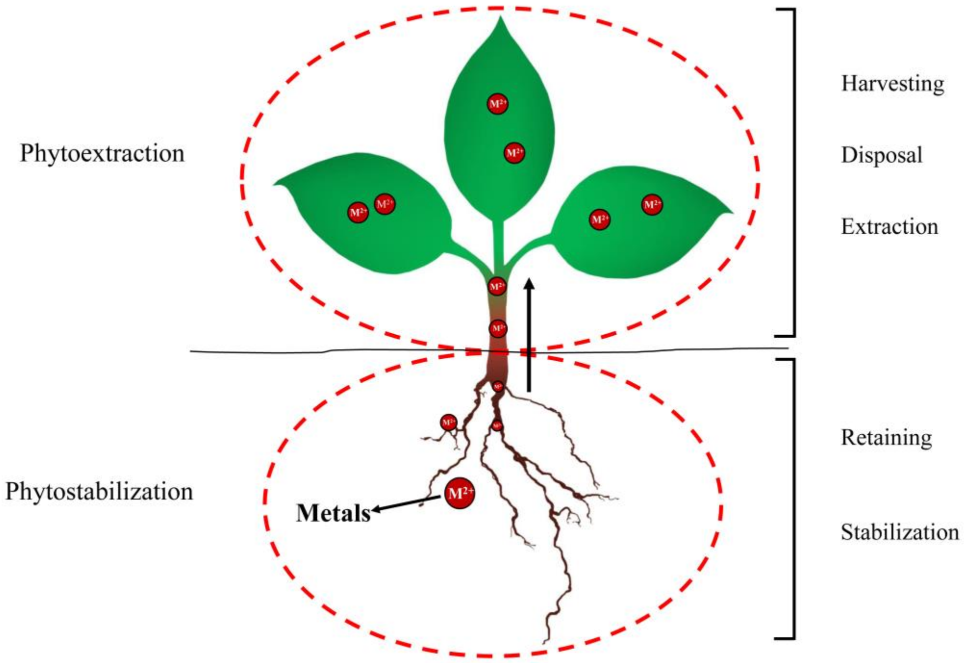

had better heavy metal removal and stabilization effects than the other native plants. Therefore, *A. breviligulata* can be selected as the preferred species for heavy metal pollution remediation in the study area.

[illegible]gure 6. Phytoextraction and phytostabilization.

5. Conclusion

The results showed that there was obvious compound heavy metal pollution in the soil around the copper-nickel tailings reservoir area. Among them, the heavy metal elements Cd, Cu, Ni, and

Cr were at the heavy pollution level; Mn and Pb were at the moderate pollution level; and Zn and As were at the slight pollution level. The PMF model results showed that the Cu and Ni came from industrial pollution sources; Cd and Cr came from atmospheric deposition and agricultural pollution sources; Pb came from traffic pollution sources; and Mn, Zn, and As came from natural sources. Compared to the normal heavy metal content in plants, the heavy metal elements in the underground and aboveground parts of the 10 desert plants surveyed exceeded the standard, indicating that heavy metal pollution in the soil posed a threat to plant growth. Among the 10 desert plants, the CEI and the CSI values for *A. breviligulata* were highest, indicating that it has a good potential to remove and stabilize a variety of heavy metals in the soil. Therefore, *A. breviligulata* had a strong comprehensive remediation potential for heavy metals in the soil around the tailings pond and it could be used to remediate heavy metal pollution in the soil. This study used fuzzy evaluation to evaluate the comprehensive extraction/stability potential of plants at a site with multiple heavy metal compound pollution. The processes used in this study successfully identified local plants with higher polymetallic remediation capacities that could be utilized in local remediation projects.

Data availability: All relevant data are within the manuscript and its Supporting Information files.

Funding: This work was funded by the National Key Research and Development Program of China (2018YFC1802903).

Competing interests: The authors have declared that no competing interests exist.

Author contribution:

Conceptualization: Jianfei Shi, Zhengzhong Jin, Zhibin Zhou.
Data curation: Jianfei Shi, Wenting Qian.
Formal analysis: Jianfei Shi.
Investigation: Jianfei Shi, Xin Wang, Xiaoliang Yang.
Methodology: Jianfei Shi.
Supervision: Jianfei Shi, Zhengzhong Jin.
Writing-Original draft: Jianfei Shi.
Writing-review & editing: Jianfei Shi, Zhengzhong Jin, Zhibin Zhou.

Reference

Adiansyah, J.S., Rosano, M., Vink, S., Keir, G., 2015. A framework for a sustainable approach to mine tailings management: disposal strategies. Journal of Cleaner Production, 108, 1050-1062.

Ashraf, S., Ali, Q., Zahir, Z.A., Ashraf, S., Asghar, H.N., 2019. Phytoremediation: Environmentally sustainable way for reclamation of heavy metal polluted soils. Ecotoxicology and environmental safety, 174, 714-727.

Cai, L.-M., Wang, Q.-S., Wen, H.-H., Luo, J., Wang, S., 2019. Heavy metals in agricultural soils from a typical township in Guangdong Province, China: Occurrences and spatial distribution. Ecotoxicology and Environmental Safety, 168, 184-191.

Cameselle, C., Chirakkara, R.A., Reddy, K.R., 2013. Electrokinetic-enhanced phytoremediation of soils:

status and opportunities. Chemosphere, 93, 626-636.

Consuelo Gazitua, M., Morgante, V., Josefina Poupin, M., Ledger, T., Rodriguez-Valdecantos, G., Herrera, C., Del Carmen Gonzalez-Chavez, M., Ginocchio, R., Gonzalez, B., 2021. The microbial community from the early-plant colonizer (Baccharis linearis) is required for plant establishment on copper mine tailings. Scientific Reports, 11.

Cui, Z., Wang, Y., Zhao, N., Yu, R., Xu, G., Yu, Y., 2018. Spatial distribution and risk assessment of heavy metals in paddy soils of Yongshuyu irrigation area from Songhua River Basin, Northeast China. Chinese Geographical Science, 28, 797-809.

El Alaoui, A., Raklami, A., Bechtaoui, N., El Gharmali, A., Ouhammou, A., Imziln, B., Achouak, W., Pajuelo, E., Oufdou, K., 2021. Use of native plants and their associated bacteria rhizobiomes to remediate-restore Draa Sfar and Kettara mining sites, Morocco. Environmental Monitoring and Assessment, 193.

Fei, X., Lou, Z., Xiao, R., Ren, Z., Lv, X., 2020. Contamination assessment and source apportionment of heavy metals in agricultural soil through the synthesis of PMF and GeogDetector models. Science of the Total Environment, 747.

Flathman, P.E., Lanza, G.R., 1998. Phytoremediation: current views on an emerging green technology. Journal of soil contamination, 7, 415-432.

Fu, S., Lu, J., 2019. Temperature-driven variation in the removal of heavy metals from contaminated tailings leaching in northern Norway. Environmental monitoring and assessment, 191, 123.

G, B., G, C., A, C., E, T., 2015. Use of native plants for the remediation of abandoned mine sites in Mediterranean semiarid environments. Bulletin of environmental contamination and toxicology, 94.

Ginocchio, R., 2000. Effects of a copper smelter on a grassland community in the Puchuncavı Valley, Chile. Chemosphere, 41, 15-23.

Guan, Q., Wang, F., Xu, C., Pan, N., Lin, J., Zhao, R., Yang, Y., Luo, H., 2018. Source apportionment of heavy metals in agricultural soil based on PMF: A case study in Hexi Corridor, northwest China. Chemosphere, 193, 189-197.

Hanfi, M.Y., Mostafa, M.Y.A., Zhukovsky, M.V., 2020. Heavy metal contamination in urban surface sediments: sources, distribution, contamination control, and remediation. Environmental Monitoring and Assessment, 192.

Hołtra, A., Zamorska-Wojdyła, D., 2020. The pollution indices of trace elements in soils and plants close to the copper and zinc smelting works in Poland's Lower Silesia. Environmental Science and Pollution Research, 27, 16086-16099.

Houben, D., Couder, E., Sonnet, P., 2013. Leachability of cadmium, lead, and zinc in a long-term spontaneously revegetated slag heap: implications for phytostabilization. Journal of Soils and Sediments, 13, 543-554.

Huang, J., Wu, Y., Sun, J., Li, X., Geng, X., Zhao, M., Sun, T., Fan, Z., 2021. Health risk assessment of heavy metal (loid) s in park soils of the largest megacity in China by using Monte Carlo simulation coupled with Positive matrix factorization model. Journal of Hazardous Materials, 415, 125629.

Huang, L., Baumgartl, T., Mulligan, D., 2012. Is rhizosphere remediation sufficient for sustainable revegetation of mine tailings? Annals of Botany, 110, 223-238.

Jeong, H., Choi, J.Y., Lee, J., Lim, J., Ra, K., 2020. Heavy metal pollution by road-deposited sediments and its contribution to total suspended solids in rainfall runoff from intensive industrial areas. Environmental Pollution, 265.

Kabata-Pendias, A., Mukherjee, A.B., 2007. Trace elements from soil to human. Springer Science &

Business Media.

Kalinovic, J.V., Serbula, S.M., Radojevic, A.A., Milosavljevic, J.S., Kalinovic, T.S., Steharnik, M.M., 2019. Assessment of As, Cd, Cu, Fe, Pb, and Zn concentrations in soil and parts of Rosa spp. sampled in extremely polluted environment. Environmental monitoring and assessment, 191, 1-14.

Karaca, O., Cameselle, C., Reddy, K.R., 2018. Mine tailing disposal sites: contamination problems, remedial options and phytocaps for sustainable remediation. Reviews in Environmental Science and Bio/Technology, 17, 205-228.

Khodaverdiloo, H., Han, F.X., Taghlidabad, R.H., Karimi, A., Moradi, N., Kazery, J.A., 2020. Potentially toxic element contamination of arid and semi-arid soils and its phytoremediation. Arid Land Research and Management, 34, 361-391.

Khorasanipour, M., 2015. Environmental mineralogy of Cu-porphyry mine tailings, a case study of semi-arid climate conditions, Sarcheshmeh mine, SE Iran. Journal of Geochemical Exploration, 153, 40-52.

Khorasanipour, M., Jafari, Z., 2018. Environmental geochemistry of rare earth elements in Cu-porphyry mine tailings in the semiarid climate conditions of Sarcheshmeh mine in southeastern Iran. Chemical Geology, 477, 58-72.

Lam, E.J., Galvez, M.E., Canovas, M., Montofre, I.L., Keith, B.F., 2018. Assessment of the adaptive capacity of plant species in copper mine tailings in arid and semiarid environments. Journal of Soils and Sediments, 18, 2203-2216.

Li, H., Xie, M., Wang, H., Li, S., Xu, M., 2020. Spatial Heterogeneity of Vegetation Response to Mining Activities in Resource Regions of Northwestern China. Remote Sensing, 12.

Li, H., Yao, J., Min, N., Liu, J., Chen, Z., Zhu, X., Zhao, C., Pang, W., Li, M., Cao, Y., 2022. Relationships between microbial activity, enzyme activities and metal (loid) form in NiCu tailings area. Science of The Total Environment, 812, 152326.

Liang, J., Chen, C., Song, X., Han, Y., Liang, Z., 2011. Assessment of heavy metal pollution in soil and plants from Dunhua sewage irrigation area. International journal of electrochemical science, 6, 5314-5324.

Liang, Y., Yi, X., Dang, Z., Wang, Q., Luo, H., Tang, J., 2017. Heavy metal contamination and health risk assessment in the vicinity of a tailing pond in Guangdong, China. International journal of environmental research and public health, 14, 1557.

Liu, K., Li, C., Tang, S., Shang, G., Yu, F., Li, Y., 2020. Heavy metal concentration, potential ecological risk assessment and enzyme activity in soils affected by a lead-zinc tailing spill in Guangxi, China. Chemosphere, 251, 126415.

Liu, Z., Hamuti, A., Abdulla, H., Zhang, F., Mao, X., 2016. Accumulation of metallic elements by native species thriving in two mine tailings in Aletai, China. Environmental Earth Sciences, 75, 1-9.

Luo, H., Wang, Q., Guan, Q., Ma, Y., Ni, F., Yang, E., Zhang, J., 2022. Heavy metal pollution levels, source apportionment and risk assessment in dust storms in key cities in Northwest China. Journal of Hazardous Materials, 422.

Luo, L., Ma, Y., Zhang, S., Wei, D., Zhu, Y.-G., 2009. An inventory of trace element inputs to agricultural soils in China. Journal of Environmental Management, 90, 2524-2530.

Lv, J., 2019. Multivariate receptor models and robust geostatistics to estimate source apportionment of heavy metals in soils. Environmental Pollution, 244, 72-83.

Lv, J., Liu, Y., Zhang, Z., Dai, J., Dai, B., Zhu, Y., 2015. Identifying the origins and spatial distributions of heavy metals in soils of Ju country (Eastern China) using multivariate and geostatistical approach.

Journal of soils and sediments, 15, 163-178.

Matanzas, N., Afif, E., Emilio Diaz, T., Gallego, J.L.R., 2021. Screening of Pioneer Metallophyte Plant Species with Phytoremediation Potential at a Severely Contaminated Hg and As Mining Site. Environments, 8.

Mendez, M.O., Maier, R.M., 2008. Phytoremediation of mine tailings in temperate and arid environments. Reviews in Environmental Science and Bio/Technology, 7, 47-59.

Meza-Figueroa, D., Maier, R.M., de la O-Villanueva, M., Gomez-Alvarez, A., Moreno-Zazueta, A., Rivera, J., Campillo, A., Grandlic, C.J., Anaya, R., Palafox-Reyes, J., 2009a. The impact of unconfined mine tailings in residential areas from a mining town in a semi-arid environment: Nacozari, Sonora, Mexico. Chemosphere, 77, 140-147.

Meza-Figueroa, D., Maier, R.M., de la O-Villanueva, M., Gómez-Alvarez, A., Moreno-Zazueta, A., Rivera, J., Campillo, A., Grandlic, C.J., Anaya, R., Palafox-Reyes, J., 2009b. The impact of unconfined mine tailings in residential areas from a mining town in a semi-arid environment: Nacozari, Sonora, Mexico. Chemosphere, 77, 140-147.

Mousavi Kouhi, S.M., Moudi, M., 2020. Assessment of phytoremediation potential of native plant species naturally growing in a heavy metal-polluted saline–sodic soil. Environmental Science and Pollution Research, 27, 10027-10038.

Mwitwa, J., German, L., Muimba-Kankolongo, A., Puntodewo, A., 2012. Governance and sustainability challenges in landscapes shaped by mining: Mining-forestry linkages and impacts in the Copper Belt of Zambia and the DR Congo. Forest Policy and Economics, 25, 19-30.

Nemerow, N.L., 1991. Stream, lake, estuary, and ocean pollution.

Padmavathiamma, P.K., Li, L.Y., 2007. Phytoremediation Technology: Hyper-accumulation Metals in Plants. Water, Air, and Soil Pollution, 184, 105-126.

Pan, L.-b., Ma, J., Wang, X.-l., Hou, H., 2016. Heavy metals in soils from a typical county in Shanxi Province, China: Levels, sources and spatial distribution. Chemosphere, 148, 248-254.

Punia, A., 2021. Role of temperature, wind, and precipitation in heavy metal contamination at copper mines: a review. Environmental Science and Pollution Research, 28, 4056-4072.

RoyChowdhury, A., Datta, R., Sarkar, D., 2018. Heavy metal pollution and remediation, Green chemistry. Elsevier, pp. 359-373.

Sarwar, N., Imran, M., Shaheen, M.R., Ishaque, W., Kamran, M.A., Matloob, A., Rehim, A., Hussain, S., 2017. Phytoremediation strategies for soils contaminated with heavy metals: modifications and future perspectives. Chemosphere, 171, 710-721.

Trifi, M., Charef, A., Dermech, M., Azouzi, R., Chalghoum, A., Hjiri, B., Ben Sassi, M., 2019. Trend evolution of physicochemical parameters and metals mobility in acidic and complex mine tailings long exposed to severe mediterranean climatic conditions: Sidi Driss tailings case (NW-Tunisia). Journal of African Earth Sciences, 158, 103509.

Wang, L.F., Bai, Y.X., Gai, S.N., 2011. Single-factor and nemerow multi-factor index to assess heavy metals contamination in soils on railway side of Harbin-Suifenhe Railway in Northeastern China, Applied Mechanics and Materials. Trans Tech Publ, pp. 3033-3036.

Wong, M.H., 2003. Ecological restoration of mine degraded soils, with emphasis on metal contaminated soils. Chemosphere, 50, 775-780.

Young, G., Chen, Y., Yang, M., 2021. Concentrations, distribution, and risk assessment of heavy metals in the iron tailings of Yeshan National Mine Park in Nanjing, China. Chemosphere, 271, 129546.

Yu, X., Zhang, Z., Feng, A., Gu, D., Zhang, R., Xia, P., Yan, W., Zhou, X., 2020. Recent history of metal

contamination in the Fangcheng Bay (Beibu Gulf, South China) utilizing spatially-distributed sediment cores: Responding to local urbanization and industrialization. Marine Pollution Bulletin, 158.

Zhang, C., Wang, X., Jiang, S., Zhou, M., Li, F., Bi, X., Xie, S., Liu, J., 2021. Heavy metal pollution caused by cyanide gold leaching: a case study of gold tailings in central China. Environmental Science and Pollution Research, 28, 29231-29240.

Zhao, X., Liu, J., Xia, X., Chu, J., Wei, Y., Shi, S., Chang, E., Yin, W., Jiang, Z., 2014. The evaluation of heavy metal accumulation and application of a comprehensive bio-concentration index for woody species on contaminated sites in Hunan, China. Environmental Science and Pollution Research, 21, 5076-5085.
