# Supplementary material for: Evaluation of soil heavy metals pollution and the phytoremediation potential of copper-nickel mine tailings ponds
Source: PLoS One. 2023 Mar 3;18(3):e0277159. doi: 10.1371/journal.pone.0277159 (PMC9983881; doi:10.1371/journal.pone.0277159)
Supplement: Attachment — Submitted filename: Review Report.docx [file pone.0277159.s001.docx]

**Review Report**

The manuscript entitled **“Heavy metal pollution characteristics of soil around a copper-nickel mine tailings pond in the northwest arid area of China and evaluation of desert phytoremediation potential”** is of good quality and written in a good way. Although the manuscript is ok, but I have added some corrections and suggestions in the PDF file to improve the manuscript. Please consider those.
