# Supplementary material for: Evaluation of soil heavy metals pollution and the phytoremediation potential of copper-nickel mine tailings ponds
Source: PLoS One. 2023 Mar 3;18(3):e0277159. doi: 10.1371/journal.pone.0277159 (PMC9983881; doi:10.1371/journal.pone.0277159)
Supplement: Attachment — Submitted filename: Response to Reviewers.docx [file pone.0277159.s003.docx]

Dear Editor,

Please find attached the corrected version of our manuscript “Heavy metal pollution characteristics of soil around a copper-nickel mine tailings pond in the northwest arid area of China and evaluation of desert phytoremediation potential”. We appreciate the constructive comments and suggestions from reviewers. These opinions help to improve academic rigor if our article. Based on their suggestion and request, we have made corrected modifications on the revised manuscript. We hope that our work can be improved again. Furthermore, we would like to show the details as follows:

Line number is according to the revised manuscript (clean version)

**Response to Reviewer 1 Comments**

**Point 1：**MNV and MTT are not given for all analyzed heavy metals. Please explain.

Response 1: Because some elements have MNV or MTT values that are too large. If displayed in a diagram, it may result in the graph not having aesthetic properties. In order to give the reader an idea of the normal range and toxicity range of each heavy metal element in the plant, I have marked in the analysis. [line 243-259]

**Response to Reviewer 2 Comments**

**Point 1：**Abstract: the abstract is too simple and some important information is not fully displayed. Abstract needs to be qualitative as well as quantitative, improve the quality of the abstract with quantitative data.

Response 1: Thanks for your comment, in the new version we have revised the abstract and added some quantitative descriptions to the abstract. [line 23-31]

**Point 2:** Line 49, G et al., 2015？

Response 2: Thanks for your comment, in the new version we have corrected. [line 45]

**Point 3:** The introduction is a bit shallow and would benefit of more depth and outlining some hypotheses, the authors need to emphasize the novelty in the introduction part. Otherwise, I believe that this article might be more suitable for local monitoring journals. And the introduction must be focused on the objectives and methods.

Response 3: Thanks for your comment, in the new version we have revised the introduction. We described the objectives of this study and the advantages of evaluation methods. [line 49-73]

**Point 4:** The quality control parts needs to be more clear: how the standard curves were constructed? what are LoD and LoQ of the methods and how they were determined? Please provide detailed information regarding the accuracy and precision of chemical analysis for every element.

Response 4: The standard curve is prepared by mixing the standard solution and diluting it with 2% nitric acid. The R value of the linear fitting curve of Cu, Ni, Cd, Cr, Zn, Pb and As is greater than or equal to 0.999, and the R value of the linear fitting curve of Mn is greater than or equal to 0.995. LoD and LoQ of the method are calculated as follows:

$$LoD=\frac{3\times sd\times A}{B}$$

$$LoQ=4\times LoD$$

Where sd is the standard deviation of 11 sample blanks, A is the dilution multiple, and B is the weighed volume. Table 1 shows the true and measured values of the plant standard sample GBW 10052a (GSB-30a). Table 2 shows the real and measured values of soil standard sample GBW07426 (GSS-12). The results showed that the measured values of plant samples and soil samples were within the true range of samples, and RSD was less than 5%. These results reflect the precision and accuracy of sample determination to some extent. Thanks for your comment, in the new version we have made it more explicitly stated out. [line 109-112]

Table1 Standard and measured values of GBW 10052a (GSB-30a).

| **GSB-30a** | **Cr** | **Mn** | **Ni** | **Cu** | **Zn** | **As** | **Cd** | **Pb** |
| --- | --- | --- | --- | --- | --- | --- | --- | --- |
| **Standard value** | **0.6±0.1** | **1170±40** | **4.4±0.3** | **13.2±0.9** | **26±3** | **0.16±0.02** | **0.2±0.02** | **1.6±0.2** |
| Measured value1 | 0.603 | 1183 | 4.62 | 13.7 | 27.8 | 0.162 | 0.215 | 1.57 |
| Measured value2 | 0.592 | 1192 | 4.62 | 13.7 | 27.6 | 0.157 | 0.196 | 1.59 |
| Measured value3 | 0.613 | 1194 | 4.64 | 14.0 | 27.7 | 0.170 | 0.213 | 1.60 |
| Measured value4 | 0.614 | 1192 | 4.48 | 13.6 | 26.1 | 0.163 | 0.218 | 1.55 |
| Measured value5 | 0.603 | 1204 | 4.49 | 13.5 | 26.1 | 0.159 | 0.194 | 1.54 |
| Measured value6 | 0.598 | 1195 | 4.48 | 13.6 | 26.5 | 0.161 | 0.211 | 1.58 |
| Measured value7 | 0.609 | 1190 | 4.37 | 13.1 | 27.1 | 0.152 | 0.198 | 1.70 |
| Measured value8 | 0.612 | 1167 | 4.30 | 12.6 | 26.0 | 0.165 | 0.209 | 1.75 |
| Mean | 0.606 | 1189 | 4.50 | 13.4 | 26.9 | 0.161 | 0.207 | 1.610 |
| SD | 0.008 | 10 | 0.12 | 0.43 | 0.78 | 0.005 | 0.009 | 0.075 |
| RSD (%) | 1.30 | 0.91 | 2.74 | 3.22 | 2.89 | 3.34 | 4.52 | 4.65 |

Table2 Standard and measured values of GBW07426 (GSS-12).

| **GSS-12** | **Cr** | **Mn** | **Ni** | **Cu** | **Zn** | **As** | **Cd** | **Pb** |
| --- | --- | --- | --- | --- | --- | --- | --- | --- |
| **Standard value** | **59±2** | **774±19** | **32±1** | **29±1** | **78±5** | **12.2±0.8** | **0.15±0.02** | **19±2** |
| Measured value1 | 57.8 | 770.3 | 32.3 | 30.1 | 76.7 | 12.73 | 0.151 | 18.6 |
| Measured value2 | 58.3 | 765.6 | 33.0 | 30.0 | 77.8 | 12.87 | 0.170 | 18.6 |
| Measured value3 | 58.6 | 772.1 | 32.6 | 30.1 | 77.9 | 12.51 | 0.160 | 18.7 |
| Measured value4 | 59.0 | 778.4 | 32.0 | 29.6 | 76.9 | 12.94 | 0.158 | 18.6 |
| Measured value5 | 57.9 | 764.2 | 31.4 | 29.8 | 73.5 | 12.91 | 0.167 | 18.0 |
| Measured value6 | 58.2 | 774.7 | 31.6 | 29.5 | 75.9 | 12.84 | 0.166 | 18.1 |
| Measured value7 | 58.7 | 781.5 | 31.4 | 28.1 | 68.3 | 12.26 | 0.160 | 18.6 |
| Measured value8 | 59.3 | 776.3 | 31.5 | 28.2 | 68.5 | 12.32 | 0.166 | 18.7 |
| Mean | 58.57 | 772.89 | 31.99 | 29.42 | 74.44 | 12.67 | 0.16 | 18.5 |
| SD | 0.54 | 6.04 | 0.59 | 0.84 | 3.97 | 0.27 | 0.01 | 0.28 |
| RSD (%) | 0.93 | 0.78 | 1.85 | 2.86 | 5.33 | 2.14 | 3.74 | 1.51 |

**Point 5:** National Soil Environment class II standard has been abolished, and you should use a new standard.

Response 5: Thank you for your comment, in the new version we have changed the reference standard. [line 188]

**Point 6:** In the "Results and Discussion" part. The authors lack (or do not show in the manuscript) the deeper knowledge in pollution assessment and source analysis of soil heavy metal.

Response 6: Thank you for your recommended articles, which have a comprehensive analysis of the sources of heavy metals. In the new version, we have conducted a deeper analysis of the sources of heavy metals in the discussion section. [line 308-349]

**Point 7:** At the end of the discussion, explore the limitations of the study.

Response 7: Thanks for your comment, in the new version we discussed the shortcomings of this study. [line 351-353]

**Point 8:** Conclusion must be rewritten based on the objectives and the main finding in this research.

Response 8: Thanks for your comment, in the new version we rewrote our conclusions based on the objectives and the main finding in this research. [line 394-406]

**Point 9:** References: it is highly recommended to use DOI.

Response 9: Thanks for your comment, in the new version we added the DOI number of references.

**Point 10:** At some parts of the manuscript, the English language is hard to follow. Please check the English by native speakers.

Response 10: Thanks for your comment. The submission has been checked by the editor whose native language is English.
